# Thermogenic diagnosis of periprosthetic joint infection by microcalorimetry of synovial fluid

**DOI:** 10.1186/s12891-020-03366-3

**Published:** 2020-06-03

**Authors:** Christian Morgenstern, Nora Renz, Sabrina Cabric, Elena Maiolo, Carsten Perka, Andrej Trampuz

**Affiliations:** 1 Charité – Universitätsmedizin Berlin, corporate member of Freie Universität Berlin, Humboldt-Universität zu Berlin, and Berlin Institute of Health, Center for Musculoskeletal Surgery (CMSC), Charitéplatz 1, D-10117 Berlin, Germany; 2 Berlin Institute of Health Center for Regenerative Therapies (BCRT), Charité – Universitätsmedizin Berlin, Berlin, Germany

**Keywords:** Periprosthetic joint infection, Microcalorimetry, Synovial fluid, Joint arthroplasty

## Abstract

**Background:**

Synovial fluid culture is the standard investigation for the preoperative diagnosis of periprosthetic joint infection (PJI). However, the culture has limited sensitivity and requires several days until result. We evaluated the value of isothermal microcalorimetry for real-time diagnosis of PJI based on heat produced by microbial growth in synovial fluid.

**Methods:**

Patients undergoing aspiration of prosthetic hip or knee joint before revision surgery were prospectively included between 2014 and 2015. The performance of microcalorimetry was compared to synovial fluid culture using McNemar’s chi-squared test. Pearson’s correlation coefficient was calculated for synovial fluid leukocyte count and microcalorimetric heat.

**Results:**

Of 107 included patients (58 knee and 49 hip prosthesis), PJI was diagnosed in 46 patients (43%) and aseptic failure in 61 patients (57%) according to institutional criteria. In 26 PJI cases (56%) the pathogen grew in synovial fluid and intra-operative cultures. The sensitivity of synovial fluid culture and microcalorimetry was both 39% and the results were concordant in 98 patients (92%). In patients with PJI, microcalorimetry missed 4 pathogens which grew in synovial fluid culture, whereas culture missed 4 pathogens detected by microcalorimetry. A linear correlation (*r* = 0.366) was found between leukocyte count and microcalorimetric heat in synovial fluid (*p* < 0.001). The median time to positivity of microcalorimetry was 9 h (range, 1–64 h) vs. 3 days for cultures (range, 1–14 days).

**Conclusion:**

Microcalorimetry of synovial fluid allows thermogenic diagnosis of periprosthetic joint infection in synovial fluid. The diagnostic performance of synovial fluid microcalorimetry is comparable to culture and delivers results considerably faster.

**Trial registration:**

This prospective study was registered on August 21, 2015 with the public clinical trial identification NCT02530229.

## Background

An early and reliable diagnosis of periprosthetic joint infection (PJI) is critical for its successful management [1–3]. Joint aspiration is the standard preoperative diagnostic procedure to diagnose PJI. However, the current diagnostic methods used in clinical routine, like microbial cultures, are time-consuming requiring at least 24 h to yield first results and often lack accuracy [1, 2]. In recent time, an increasing number of new diagnostic methods for fast and reliable diagnosis of infection are being investigated to overcome the limitations of microbial cultures, such as synovial fluid leukocyte count [4], multiplex polymerase chain reaction (PCR) [5], leukocyte esterase [6], alpha-defensin [7–10] and next generation sequencing [11, 12]. However, these tests have limitations including technical complexity, long processing time, insufficient sensitivity or specificity and inability to identify the causing pathogen.

Isothermal microcalorimetry is a novel method for real-time detection of growth-related heat production of reproducing microorganisms in biological fluid [13]. This highly sensitive and rapid, real-time detection method has recently shown promising results using synovial fluid for the diagnosis of septic arthritis [14, 15], with a sensitivity of up to 89% and a specificity of 99%. Similarly, microcalorimetry of sonication fluid was useful in diagnosing PJI [16] with a considerably faster detection time (6 h) than conventional microbial cultures.

Microcalorimetry of synovial fluid has not yet been evaluated for the diagnosis of PJI. The aim of this prospective study is to assess the diagnostic performance and speed of microcalorimetry in the diagnosis of PJI of the hip and knee and to compare it to current standard diagnostic methods employed in clinical routine, including synovial fluid leukocyte count and microbial culture. We hypothesized that microcalorimetry of synovial fluid could improve and accelerate the diagnosis of PJI.

## Methods

### Study design

Our institutional review board issued its approval for this prospective study (EA1/306/14) and written informed consent was obtained for all patients. This study is compliant with the Declaration of Helsinki and was registered under the public clinical trial identification NCT02530229 (https://clinicaltrials.gov/ct2/show/NCT02530229).

### Study population

Patients aged 18 years and older presenting with a painful prosthesis underwent joint aspiration between December 2014 and November 2015 in a tertiary healthcare facility as part of routine preoperative diagnosis. Patients with an aspirated synovial fluid volume below 5 ml, joint aspiration within 6 weeks of the last surgery of the affected joint, periprosthetic fracture and prothesis dislocation were excluded from this study.

### Study definitions

At least one of the following criteria were necessary for diagnosing PJI, as used in other studies [5, 9, 10, 17–19]: (i) visual identification of macroscopic purulence around the prosthesis, (ii) a visible sinus tract, (iii) an elevated synovial fluid leukocyte count (> 2000 leukocytes/μl) or differential (> 70% granulocytes) [3, 4], (iv) positive culture of synovial fluid, periprosthetic tissue or culture of sonication, (v) histopathologic inflammation with ≥2 granulocytes per high-power field in periprosthetic tissue (corresponding type II or III according to Krenn and Morawietz [20]).

Positivity of synovial fluid culture was considered for a confined microbial organism in solid media culture. For enrichment broth, positivity was contemplated only for high-virulent pathogens. Positivity for culture of periprosthetic tissue was defined for (i) growth of one or more highly virulent pathogens (*Enterobacter*spp., *Streptococcus*, *Candida*, *Staphylococcus aureus*) or (ii) growth of at least two low virulent organisms (coagulase-negative staphylococci, *Cutibacterium* spp., enterococci, and pathogens of the regular skin microbioma) [21]. Growth of a high-virulent pathogen in sonication fluid of one or more colony forming unit (CFU)/ml or growth of > 50 CFU/ml of a low-virulent pathogen were required to confirm positivity for sonication [22].

### Joint aspiration

Asepsis was applied to the skin before joint aspiration. The corresponding joint was aspirated with a sterile 18-gauge spinal needle in consonance with aseptic directives. Synovial fluid was aspirated into a 10-ml syringe. The needle was relocated inside the joint without trespassing the skin if the initial joint aspiration was void.

### Synovial fluid conventional tests

A pediatric blood culture bottle (BacTec PedsPlus/F, Beckton Dickinson&Co., IE) with at least 1 ml of synovial fluid was employed for culture (at 37 °C) and monitored for 14 days (or until positivity). For gram stain an additional minimum of 1 ml was applied. Further explorations comprised Schaedler, chocolate and blood agar plates, and thioglycolate broth. Aerobic and anaerobic cultures at 37 °C were monitored daily for 7 and 14 days, respectively. Exploration for pyrophosphate and urate crystals and inflammatory cells was performed with polarization microscopy on an additional 1 ml of synovial fluid. An ethylenediaminetetraacetic acid (EDTA) vial was filled with at least 1 ml of synovial fluid to determine leukocyte count and differential. 10 μl of hyaluronidase (Sigma-Aldrich, DE) was applied on clotted samples at room temperature conditions for a 10 min-period.

### Microcalorimetry of synovial fluid

**Fig. 1:**
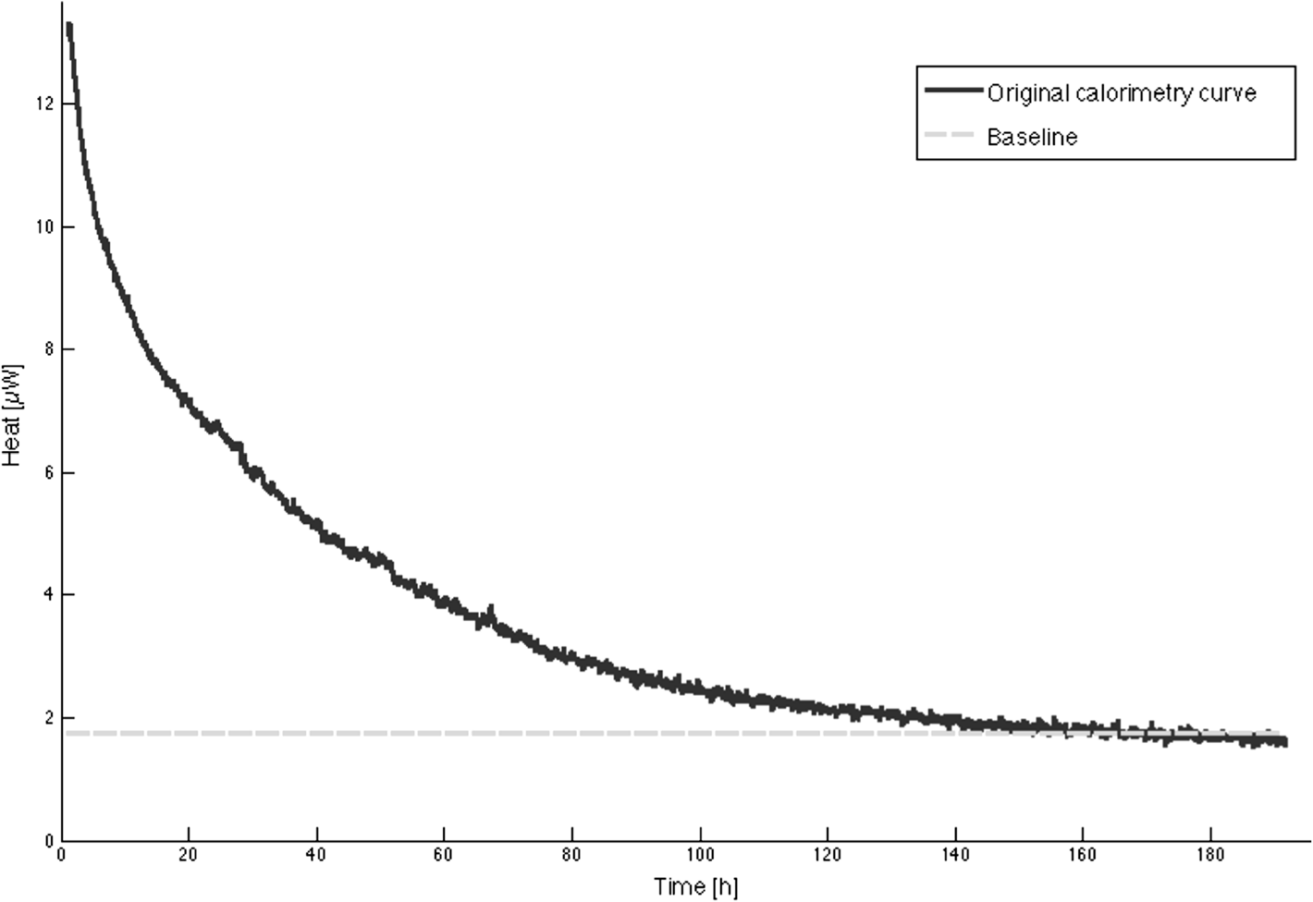
Example of a microcalorimetry curve of an aseptic patient. Note the characteristic inverse exponential decay of heat until reaching baseline (dashed line)

For microcalorimetric analysis, an aliquot of 1 ml synovial fluid was inoculated into a 4 ml microcalorimetry vial pre-filled with 2 ml of tryptic soy broth. The vial was inserted into an isothermal microcalorimeter with 48 measuring channels (model 3102 TAM III, TA Instruments, New Castle, DE, USA). According to the manufacturer, the sensitivity of the microcalorimeter is 0.225 μW. First, an equilibration process was performed for 15 min in order to reach 37 °C and another 45 min to get accurate measurement of heat flow. Then, heat production was continuously measured for a minimum of 5 days, expressed in μWatts over time. Calorimetry heat peak values were measured as the difference between the maximum heat value of the curve and its baseline value (Fig. 1). Baseline was defined as the heat value approximated by the asymptote at the end of the timeline. The time to positivity for microcalorimetry was determined and was defined as the time elapsed between insertion of the ampoule into the microcalorimeter and a rising heat curve reaching a minimum threshold of 10 μW compared to baseline [14, 15]. Upon termination of microcalorimetry testing, aerobic and anaerobic agar plating was performed with the remaining microcalorimetric probe.

### Intraoperative diagnostic tests

Cultures and histopathological examination were performed on periprosthetic tissue obtained in revision surgery. For intra-operative diagnostics periprosthetic tissue samples were collected by the surgeon and sent in for culture (at least 4 samples) and histopathologic examination (at least 1 sample). Similar to the aforementioned processing of synovial fluid, homogenization and culture of periprosthetic tissue samples was performed on aerobic and anaerobic agar plates and thioglycolate broth. Microbial pathogens were automatically determined according to standard procedure (VITEK 2, BioMérieux, FR). Sonication of the extracted implant was performed following standard technique [18].

### Statistical analysis

The diagnostic methods were evaluated with McNemar’s Chi-squared test (values at *p* < 0.05 were considered statistically significant). For aseptic calorimetry curves, we explored the association between leukocyte count in synovial fluid and heat production with Pearson’s correlation coefficient r (strong correlation for r > 0.8, modest for *r* = 0.5–0.8, and weak for *r* < 0.5). The relationship between leucocyte count and peak heat production was explored with linear regression.

## Results

### Demographics and infection data

Of 135 evaluated patients, 107 patients were included in this study. Excluded were 11 patients with insufficient synovial fluid (of whom 10 had a hip and 1 knee prosthesis), 5 with aspiration within 6 weeks of surgery, 4 patients with periprosthetic fracture and 8 patients with a prosthesis dislocation. The demographic characteristics of included patients is shown in Table 1. A total of 69 patients (65%) underwent revision surgery. For these patients, additional intra-operative samples were obtained for microbiological and histopathological analysis, as well as sonication of the extracted prosthesis.

**Table 1:** Demographic characteristics of 107 study patients

Characteristic	Periprosthetic joint infection	Aseptic failure	Total
Number of patients	46 (43%)	61 (57%)	107 (100%)
Hip prosthesis	21	37	58
Knee prosthesis	25	24	49
Age (median, range), years	72 (42–84)	68 (36–87)	71 (36–87)
Males	19	22	41 (38%)

Of 46 (43%) patients diagnosed with PJI, the infecting microorganism was identified in 26 (56%) cases. The pathogen was isolated in 18 (39%) PJI cases by synovial fluid culture only. The pathogen was detected in an additional 8 cases with the addition of periprosthetic tissue and sonication fluid cultures. Of these 8 patients with negative synovial fluid cultures, 6 cases had positive periprosthetic tissue cultures, 4 cases had positive sonication cultures and 2 cases had both. Hence, 26 (56%) PJI cases had any positive culture.

For 20 PJI cases (44%), no pathogen was identified as all (synovial fluid, periprosthetic tissue and sonication fluid) cultures remained negative.

Out of the 18 identified pathogens in synovial fluid culture, the most recurrent causative organisms detected were *Streptococcus* spp. in 8 (44%) cases, coagulase-negative staphylococci in 3 (17%) cases, *Escherichia coli* in 3 (17%) and *Enterococcus* spp. in 3 (17%) cases. No polymicrobial infections were detected by synovial fluid culture.

### Results of microbiological methods

**Fig. 2:**
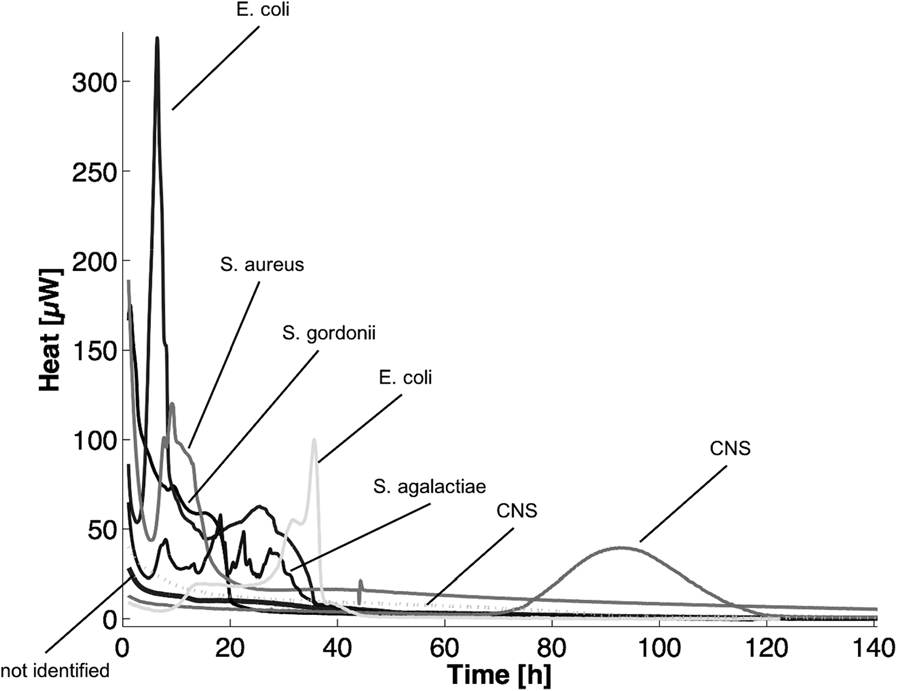
Examples of microcalorimetric signals of patients with PJI. The microorganisms mentioned in the legend were found in synovial fluid culture. CNS: coagulase-negative staphylococci

The results for the diagnostic methods are listed in Table 2 and positive calorimetry curves are shown in Fig. 2. Synovial fluid leukocytes (absolute leukocyte count or percentage of granulocytes) was the most sensitive diagnostic method for the diagnosis of PJI (80%), followed by periprosthetic tissue histopathology (74%). The sensitivity of synovial fluid microcalorimetry and culture was both 39%. The overall accuracy of all pre- and post-operative cultures (synovial fluid, periprosthetic tissue and sonication fluid) was 81%. The median time until positivity for microcalorimetry was 9 h (range, 1–64 h).

**Table 2:** Comparison of diagnostic tests for the diagnosis of periprosthetic joint infection in 107 patients

Positive test	No. patients	Aseptic failure (***n*** = 61)	PJI (***n*** = 46)	Sensitivity % (CI 95%)	Specificity % (CI 95%)	PPV % (CI 95%)	NPV % (CI 95%)	Accuracy %
**Positive preoperative test**								
Leukocyte count or percentage of granulocytes	72	2/37	28/35	80 (67–94)	95 (87–101)	93 (84–102)	83 (72–95)	88
Synovial fluid culture	107	0	18	39 (25–53)	100 (100)	100 (100)	69 (59–78)	74
Synovial fluid microcalorimetry	107	1	18	39 (25–53)	98 (95–102)	95 (85–105)	68 (59–78)	73
**Positive intraoperative test**								
Periprosthetic tissue histology	48	0/21	20/27	74 (58–91)	100 (100)	100 (100)	75 (59–91)	85
Periprosthetic tissue culture	61	0/28	15/33	46 (29–63)	100 (100)	100 (100)	61 (47–75)	71
Sonication fluid culture	28	0/11	10/17	59 (35–82)	100 (100)	100 (100)	61 (39–84)	75

Where the denominator is shown, data were not available for all cases. *PJI* periprosthetic joint infection, *PPV* positive predictive value, *NPV* negative predictive value, *95% CI* 95% confidence interval

### Concordance of synovial fluid culture and microcalorimetry

In 98 of 107 patients (92%), synovial fluid culture and microcalorimetry showed concordant results, including 38 PJI and 60 aseptic cases (Table 3). In patients with PJI, microcalorimetry missed 4 cases where cultures grew the pathogen, including *E. faecalis* (2 isolates), coagulase-negative staphylococci and *E. coli* (1 isolate each). In contrast, microcalorimetry was positive for 5 cases with negative synovial fluid culture (Table 4). Culture of synovial fluid after microcalorimetric measurement was performed in 68 cases (64%) (Table 4). Microcalorimetry was positive in one aseptic failure case with negative synovial fluid culture. The agar plating of this microcalorimetric probe was negative and the case was considered as false-positive.

**Table 3:** Concordance of 107 detected microorganisms by synovial fluid culture and microcalorimetry

Diagnostic test		Periprosthetic joint infection (***n*** = 46)		Aseptic failure (***n*** = 61)	
Culture	Microcalorimetry				
+	+	14	*S. aureus* (1) *E. coli* (2) *Streptococcus* spp. (8)^*a*^ *E. faecalis* (1*)* CNS (2)	0	
–	–	24		60	
**Total concordant**		**38**		**60**	
+	–	4	*E. faecalis* (2) CNS (1) *E. coli* (1)	0	
–	+	4	*Pseudomonas aeruginosa* (1)^b^ CNS (1)^b^	1	
**Total discordant**		**8**		**1**	

*CNS* coagulase-negative staphylococci, *primers not included in the PCR test kit

^a^*S. agalactiae* (*n* = 4), *S. mitis/oralis* (*n* = 3), *S. gordonii* (*n* = 1)

^b^ In one patient, two organisms were isolated during revision surgery

**Table 4:** Discordant results with positive synovial fluid microcalorimetry in patients with negative synovial fluid culture

Pat. No.	Joint	Class	Synovial fluid leukocyte count (/μl) / granulocytes (%)	Synovial fluid culture	Periprosthetic tissue culture^**a**^ (No. positive / No. all samples)	Sonication fluid culture^**a**^	Periprosthetic tissue histology^**a**^	Synovial fluid culture after microcalorimetry
1	Knee	PJI	20,354/69^a^	Negative	*Pseudomonas* (3/4)	CNS	Infection	ND
2	Hip	PJI	30,143/99 ^b^	Negative	ND	ND	ND	*Positive*
3	Hip	PJI	35,910/95 ^b^	Negative	ND	ND	ND	Negative
4	Hip	PJI	2202/72^a^	Negative	Negative (0/5)	Negative	Wear	Negative
5	Hip	Aseptic	ND	Negative	Negative (0/5)	ND	Wear	Negative

*CNS* coagulase-negative staphylococci, *PJI* periprosthetic joint infection, *ND* not done

^a^ Results from specimen harvested during revision surgery

^b^ Results from preoperative synovial fluid aspiration

### Association of synovial fluid leukocyte count and microcalorimetry

**Fig. 3:**
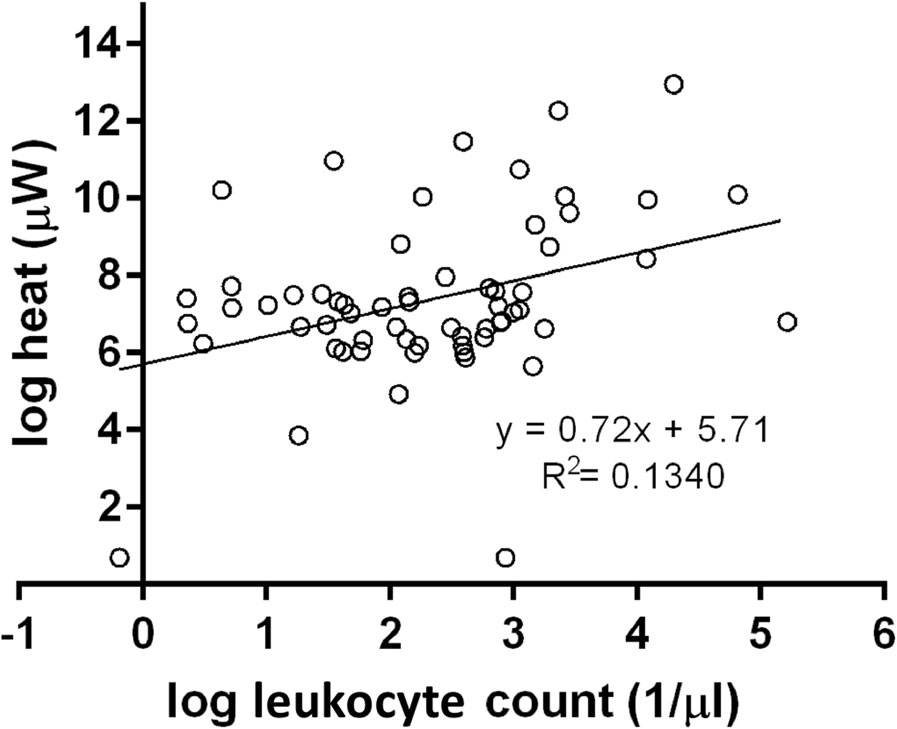
Positive linear association of peak heat values measured with microcalorimetry with leukocyte count in synovial fluid (*p* = 0.003) for aseptic calorimetry curves

Pearson’s correlation coefficient for log leucocyte count and log peak heat production showed a weak linear correlation coefficient of *r* = 0.366 with a 95% confidence interval 0.130–0.567 and a significance of *p* < 0.001 (Fig. 3). Linear regression showed that a 1% increase in leukocyte count is associated with a 0.72% increase in peak heat production (*p* = 0.003).

## Discussion

Non-microbiological tests such as synovial fluid leukocyte count and periprosthetic tissue histopathology demonstrated high sensitivity for diagnosing PJI. However, these methods are unable to identify the causing pathogen and its antimicrobial susceptibility. Moreover, periprosthetic tissue histopathology is not feasible in pre-operative diagnostics as representative samples of periprosthetic tissue are difficult to obtain by arthroscopy [1, 23].

To our knowledge, this is the first report on microcalorimetry of synovial fluid for the diagnosis of PJI. Microcalorimetry allows real-time analysis of a pathogen’s heat production curve. The heat production profile can be used for rapid identification of the corresponding pathogen [24]. Hence, microcalorimetry could be used as a fast screening method for PJI. Moreover, positive microcalorimetry probes amplify the pathogen during processing, which accelerates the pathogen identification by other methods, such as Matrix-Assisted Laser Desorption/ionization-Time-Of-Flight mass spectrometer (MALDI-TOF) [25] and antimicrobial susceptibility testing.

Microcalorimetry was positive for 5 cases with negative synovial fluid culture. Given the clear clinical manifestations for cases 1, 2 and 3 (Table 4), synovial fluid culture is most likely false-negative for these cases. Hence, microcalorimetry seems to be more sensitive in cases with a high leukocyte count than conventional culture.

Microcalorimetry was positive in one aseptic failure case with negative synovial fluid culture. The agar plating of this microcalorimetric probe was negative and the case was considered a false-positive. The misclassification of this case may be associated with a misinterpreted heat spike. The source of the heat spike could be related to increased synovial fluid leukocyte count, as a high leukocyte count initially produce a heat peak as has been demonstrated here (Fig. 3).

There are several limitations of our study. First, the sensitivity of microcalorimetry was lower (39%) than in previously reported results [1–4, 6, 23]. This observation may be explained by previous antibiotic treatment in most patients, which were transferred to our hospital from elsewhere. This may also explain that for 44% of the PJI cases all cultures were negative, and the PJI diagnosis was made by non-microbiological tests. Second, in some specific cases pathogens have been identified with the help of their microcalorimetry curve only [24]. However, microcalorimetric characterization of the most common pathogens is still pending. In future work, this could probably be implemented with automatic microcalorimetric pattern analysis. Finally, increased leukocyte counts due to aseptic cause in synovial fluid (i.e. crystal-induced arthritis) may lead to significant heat production due to leukocytes rather than bacteria (Fig. 3). In this study, we have insufficient cases with aseptic inflammatory conditions to evaluate the specificity of the microcalorimetry.

## Conclusion

Microcalorimetry of synovial fluid allowed thermogenic diagnosis of periprosthetic joint infection in synovial fluid, showing a similar sensitivity and specificity than culture but providing faster results (median of 9 h vs. several days, respectively). However, given its limited sensitivity, microcalorimetry presents restrictions similar to culture for the diagnosis of PJI. Hence, with further improvement of its performance, microcalorimetry could complement conventional cultures and support rapid, real-time decisions in orthopedic-device related infections.

## Data Availability

The datasets used and/or analyzed during the current study are available from the corresponding author on reasonable request.

## Abbreviations

CFU: Colony forming unit
MALDI-TOF: Matrix-Assisted Laser Desorption/ionization-Time-Of-Flight mass spectrometer
NPV: Negative predictive value
PCR: Polymerase chain reaction
PJI: Periprosthetic Joint Infection
PPV: Positive predictive value
